# Polytetrafluoroethylene (PTFE) Tape as a Screw-Access Sealing Material in Implant-Supported Crowns

**DOI:** 10.7759/cureus.102619

**Published:** 2026-01-30

**Authors:** Mario D Ramos

**Affiliations:** 1 Preclinical Faculty, College of Dental Medicine - Illinois, Midwestern University, Downers Grove, USA

**Keywords:** composite resin, dental-implantology, endosseous dental implants, fixed prosthodontics, polytetrafluoroethylene (ptfe), restorative dentistry

## Abstract

A clear consensus is lacking in the literature regarding the optimal material for sealing the screw‑access channel in implant‑supported restorations. Commonly used materials, including cotton pellets, polyvinyl siloxane, and gutta‑percha, are selected largely based on clinician preference rather than evidence‑based guidelines. Polytetrafluoroethylene (PTFE) tape has emerged as a practical alternative, offering several advantages for sealing the screw‑access channel in screw‑retained implant crowns. PTFE is inexpensive, readily adaptable, easy to manipulate, and maintains dimensional stability even after sterilization. Furthermore, it facilitates efficient screw access during maintenance procedures without compromising the protection of the abutment and prosthetic screw.

This technical report describes a straightforward technique in which PTFE tape is utilized as a screw‑access sealing material for screw‑retained implant‑supported crowns, providing clinicians with a reliable and retrievable option for prosthetic management.

## Introduction

Implant‑supported crowns can be delivered using either cement-retained or screw-retained designs. Although the advantages of each approach continue to be debated in the literature, clinical trends have increasingly favored screw‑retained restorations [1]. This shift reflects growing awareness of the complications associated with excess cement and the desire for improved retrievability and long‑term maintenance [2-4]. Cement‑retained crowns offer several advantages, including reduced risk of screw loosening [2], improved esthetics due to the absence of a screw‑access channel [2,3,5], enhanced preservation of the restoration's structural integrity [3], and the possibility of achieving a near‑ideal passive fit [2]. Nevertheless, cement‑retained restorations may induce peri‑implant inflammation when excess luting agent is not completely removed [4,6]. Wilson, using a dental endoscope, identified cement remnants as the etiologic factor in over 80% of implant sites exhibiting peri‑implantitis, with more than 70% of sites showing resolution once the residual cement was eliminated [4]. In contrast, screw‑retained implant crowns eliminate the risk of complications associated with residual cement, as the prosthetic screw connects directly to the implant fixture. Furthermore, screw‑retained restorations offer a significant advantage in that they allow more predictable outcomes over the last two decades [1] and conservative retrieval when maintenance or repair is required [2,7,8].

Although implant therapy exhibits high survival rates (97.7% at five years and 94.9% at 10 years) [9], and mechanical complication rates ranging from 95% for fixed prostheses to 95%-100% for overdentures [10], late biological complications remain a major concern. It is important to note that when comparing survival rates reported in publications before and after 2000, there appears to be an overall increase from 93.5% to 97.1% [1]. Specifically, survival rates for cemented prostheses rose from 95.2% to 97.9%, while screw‑retained options demonstrated a substantial improvement, increasing from 77.6% to 96.8% [1]. Occlusal factors and the complex oral microbiome have been strongly associated with peri‑implant tissue breakdown [5,9-11]. Peri‑implantitis affects approximately one in five patients receiving implants and one in 10 endosseous implants placed [12]. Consequently, preventive measures, including risk factor modification and meticulous biofilm control, are essential components of ongoing patient management [11,12]. 

Biofilm formation plays a critical role in implant success [13]. Evidence suggests that biofilm can develop around implant fixtures in a manner similar to natural dentition [11,13-16]. Both qualitative and quantitative plaque accumulation have been linked to long‑term implant outcomes [11,13,17,18], and multiple risk factors and diagnostic indicators for peri‑implant mucositis and peri‑implantitis have been reported [11]. Bacterial penetration (microleakage) may occur at the implant‑abutment interface, between the implant and the crown, and at the surfaces of these components [13,17,19]. Microleakage may also originate at the interface between the crown and the screw‑access sealing material as a result of polymerization shrinkage. Research evaluating microleakage pathways in implant‑supported crowns has shown that bacterial penetration occurs more frequently through the screw‑access channel than through the implant‑abutment interface [13,14,20]. A systematic review and meta‑analysis by Mao and collaborators concluded that bacterial microleakage commonly occurred under dynamic loading in vitro; however, the evidence regarding the performance of different sealing materials was insufficient to draw definitive conclusions [21]. The selection of a screw‑access sealing material [10,22,23] or cleaning agent [24] may therefore influence the long‑term success of implant restorations, yet the literature remains limited, and clinical decisions often depend on clinician preference and experience [23].

Several materials have been proposed for sealing the screw‑access channel in implant-supported crowns. Cotton pellets [14,25-27] are widely used due to their low cost and ability to be sterilized, though they can be difficult to remove and may produce unpleasant odors upon retrieval [18]. Gutta‑percha [14,23,28-30] is easy to manipulate but cannot be sterilized. Polyvinyl siloxane (PVS) [14,23,28,29] can be sterilized without adversely affecting its physical properties [31-33].

Polytetrafluoroethylene (PTFE), commonly known as Teflon tape, is composed of viscoelastic polymers layered with tetrafluoroethylene. It has been widely used in dentistry for various applications [34-47], though its use in implant dentistry has been less extensively documented. Elter and collaborators [44] compared biofilm formation on titanium and PTFE abutment surfaces and found significantly reduced biofilm accumulation on PTFE. Moráguez and Belser [45] later described the clinical use of PTFE as a screw‑access sealing material in implant prosthetics.

A national survey by Tarica and researchers [48] reported that cotton pellets, PVS, and gutta‑percha were the most frequently used materials for sealing screw‑access channels. Park and collaborators [22] conducted the first investigation comparing microleakage among different sealing materials in internal‑connection implants and found the following leakage ranking (greatest to least): gutta‑percha, PVS, silicone‑based material, and cotton pellets; gutta‑percha and PVS did not differ significantly. In another in vitro study, Paranjpe and colleagues [49] reported that PTFE yielded no bacterial contamination, whereas cotton pellets showed high levels of contamination. Similarly, do Nascimento and colleagues found that PTFE combined with resin composite produced the least amount of leakage as evidenced by presenting the lowest bacterial counts, whereas cotton pellets and resin demonstrated the highest leakage [27]. It should be noted, however, that long-term clinical outcome data regarding the in vivo use of PTFE as a sealing material remain limited.

The purpose of this technical report is to present a technique utilizing polytetrafluoroethylene (PTFE) tape as a screw‑access sealing material for screw‑retained implant‑supported crowns.

## Technical report

Laboratory procedures

After successful try‑in, 9.6% hydrofluoric acid (Ivoclar Vivadent, Amherst, NY, USA) was applied to the occlusal and axial walls of the screw‑access channel for one minute and 30 seconds to roughen the ceramic surface and enhance micromechanical retention. The area was thoroughly rinsed with water and dried completely.

Intraoral procedures 

Rubber dam isolation was achieved, and the retainer surface was carefully coated with PTFE (Teflon) tape to prevent contact between metallic instruments and the endosseous implant.

The screw‑retained crown was torqued according to the manufacturer’s recommendations, followed by irrigation of the screw‑access channel with 0.12% chlorhexidine gluconate (Peridex, 3M ESPE, St. Paul, MN, USA) delivered with a plastic syringe [24].

**Figure 1:**
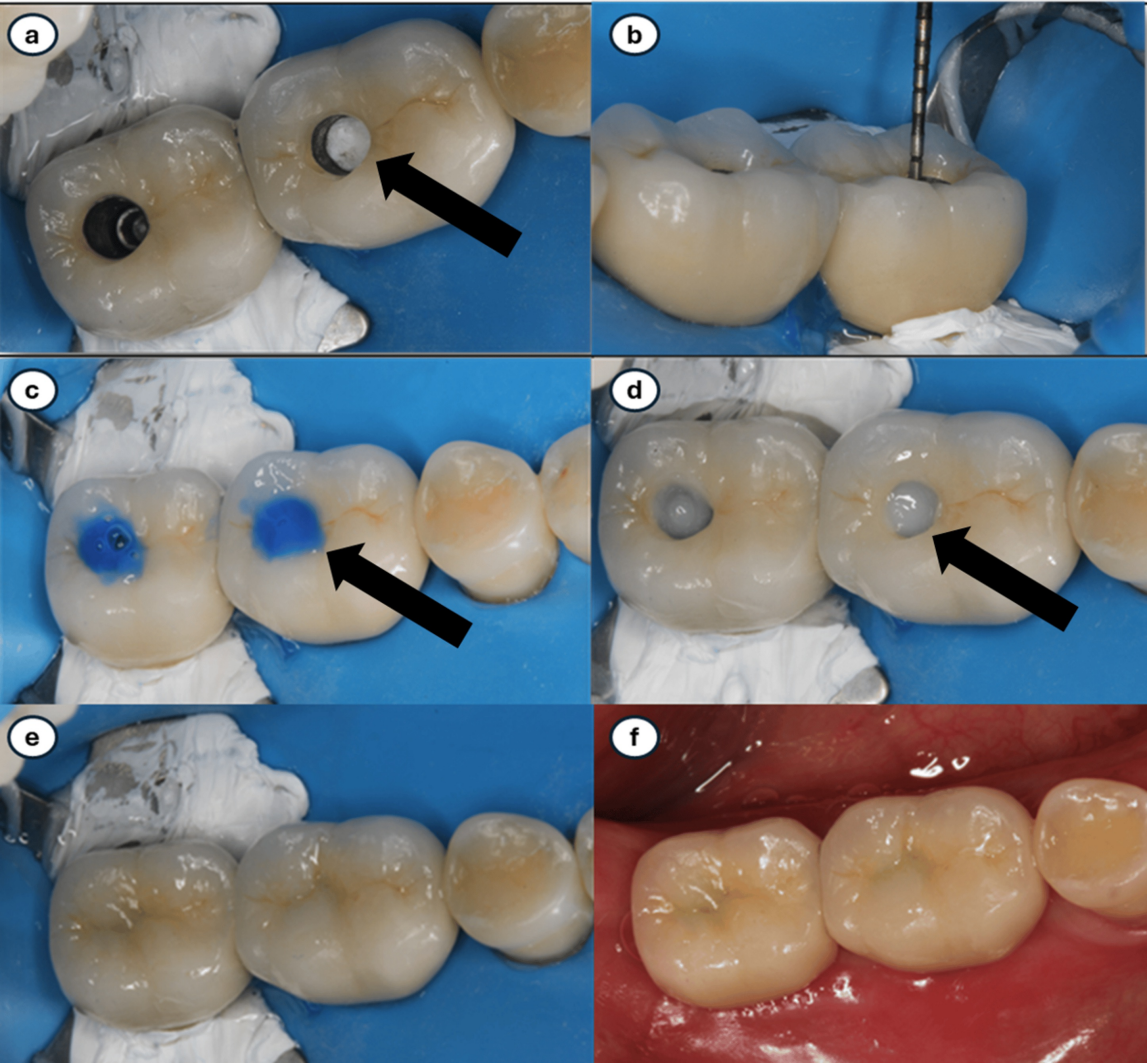
(a) Teflon tape condensed within the screw-access channel. (b) Two to three millimeters were left to allow space for the composite resin. (c) Phosphoric acid was applied to the surrounding areas of the access channel entry. (d) Composite resin was added incrementally and cured, starting with the opaque shade. (e) Care was taken to mimic the existing anatomical features. (f) After confirming occlusion, final polishing was performed.

The screw‑access channel was then filled with PTFE tape using a ½ condenser (Hu‑Friedy, Chicago, IL, USA), applying condensation pressure approximating 10 MPa, similar to amalgam condensation (Figure 1a). Approximately 2-3 mm of occlusal space was intentionally preserved for the definitive direct restoration (Figure 1b).

The occlusal and axial walls of the channel were etched with 35% phosphoric acid (Ultra‑Etch; Ultradent Products, South Jordan, UT, USA) for 15 seconds, rinsed with water for 10 seconds, and air‑dried for five seconds (Figure 1c). A silane coupling agent (Monobond S, Ivoclar Vivadent, Amherst, NY, USA) was applied for 60 seconds and gently air‑thinned with high‑speed suction. 

A thin layer of bonding agent (Optibond FL, Kerr, Brea, CA, USA) was applied and light cured in accordance with manufacturer guidelines. Composite resin (Filtek Supreme, 3M ESPE) was placed incrementally, beginning with an opaque shade to mask the PTFE and followed by additional shades selected to harmonize with the ceramic (Figure 1d). Each increment was light cured for 20 seconds according to manufacturer's recommendations (Figure 1e).

Occlusion was checked, and the composite restoration was polished using standard polishing techniques (Figure 1f).

## Discussion

Selection of an appropriate sealing material for the screw‑access channel in screw‑retained implant restorations remains clinically relevant because of its impact on microleakage, prosthesis retrievability, and long‑term peri‑implant tissue stability. Evidence indicates that bacterial penetration through the access channel may represent a more significant contamination pathway than leakage at the implant‑abutment interface [13,14,20], reinforcing the need for a material that reliably minimizes ingress while permitting predictable maintenance access.

Cotton pellets remain widely used due to their availability and low cost [14,25-27], yet they present well‑documented limitations, including odor development and difficulties in removal [18]. Other materials such as gutta‑percha and PVS have shown inconsistent performance. PVS can be sterilized without property degradation [31-33], but studies report mixed results regarding its ability to prevent bacterial leakage [22]. A systematic review by Mao and collaborators emphasized that most microleakage studies rely on in vitro models and heterogeneous methodologies, limiting the ability to identify a clearly superior sealing material [21]. Consequently, material selection in clinical practice has often depended on individual preference and experiential judgment rather than strong evidence.

More recent investigations have brought attention to PTFE tape as a viable alternative. Paranjpe and researchers reported an absence of bacterial contamination when PTFE was used beneath provisional restorations, whereas cotton pellets demonstrated substantial microbial penetration [49]. Likewise, do Nascimento and colleagues found that PTFE combined with a resin composite resulted in lower bacterial counts compared with other combinations, particularly cotton pellets with resin [27]. These findings suggest that PTFE may provide superior resistance to microleakage relative to other sealing materials.

Beyond microleakage itself, the biological implications of sealing‑material choice warrant careful consideration. Peri‑implantitis represents one of the most common biological complications, affecting up to one in five implant patients and one in ten implants [12]. Residual cement remains a leading etiologic factor [4], strengthening the rationale for screw‑retained restorations, which eliminate cement‑related risks entirely. Effective sealing of the screw‑access channel may further support peri‑implant health by reducing plaque accumulation and limiting bacterial colonization of prosthetic components [13,17,19].

PTFE also offers advantageous surface characteristics. Elter and researchers demonstrated significantly reduced biofilm formation on PTFE compared with titanium [44], suggesting that the material inherently limits microbial adherence. Combined with its low cost, ease of manipulation, dimensional stability, and predictable removability, PTFE presents an appealing option for clinicians seeking a biologically favorable and retrievable sealing material.

The technique presented in this technical report builds upon the foundational work of Moráguez and Belser [45], expanding the clinical application of PTFE through laboratory surface conditioning, implant‑safe handling, and a layered restorative approach designed to enhance retention, minimize microleakage, and maintain esthetics. This protocol remains simple, reproducible, cost‑effective, and highly compatible with long‑term maintenance of screw‑retained implant restorations.

## Conclusions

PTFE tape represents a practical and biologically favorable option for sealing the screw‑access channel in the screw‑retained implant‑supported crowns. Its ease of manipulation, low cost, dimensional stability, and predictable retrievability make it a compelling alternative to more traditional spacer materials. Current evidence indicates that PTFE may reduce bacterial contamination and microleakage when compared with commonly used materials such as cotton pellets and gutta‑percha, thereby supporting long‑term peri‑implant tissue health. The technique described in this technical report provides a simple, reproducible, and efficient method for incorporating PTFE into routine implant prosthodontic procedures. Adoption of this approach may enhance maintenance protocols, improve clinical outcomes, and contribute to reducing the biological complications associated with implant‑supported restorations.
